# Association between educational level and acceptance of antenatal respiratory syncytial virus vaccination among Saudi women: a cross-sectional study

**DOI:** 10.3389/fped.2026.1900895

**Published:** 2026-07-17

**Authors:** Amer Alshengeti, Maha A. Rizq, Afnan N. Almuhammdi, Fatima Tajelsir Ali, Fatema Abdulkarim Saleh, Faisal N. Kordy, Hala Mohammed Mousa, Abdulsalam Alawfi, Ibrahim Sandokji

**Affiliations:** 1 Section of Pediatrics, Department of Women’s and Children’s Health, College of Medicine, Taibah University, Al-Madinah, Saudi Arabia; 2 Infection Prevention and Control Department, Prince Mohammed Bin Abdulaziz Hospital, Ministry of National Guard-Health Affairs, Al-Madinah, Saudi Arabia; 3 Department of Pediatrics, Prince Mohammed Bin Abdulaziz Hospital, Ministry of National Guard-Health Affairs, Al-Madinah, Saudi Arabia; 4 Faculty of Medicine and Surgery, National University of Sudan, Khartoum, Sudan; 5 College of Medicine, Taibah University, Al-Madinah, Saudi Arabia; 6 Division of Infectious Diseases, Department of Pediatrics, King Salman Medical City, Al-Madinah, Saudi Arabia; 7 Department of Obstetrics and Gynecology, King Salman Medical City, Al-Madinah, Saudi Arabia

**Keywords:** acceptance, awareness, maternal immunization, respiratory syncytial virus, Saudi Arabia, vaccine

## Abstract

**Introduction:**

Recently, a respiratory syncytial virus (RSV) vaccine was approved and recommended for pregnant women in Saudi Arabia. Data are needed regarding pregnant women's awareness of RSV infection and their acceptance of the RSV vaccine. Therefore, in this study, we aimed to assess the awareness and acceptance of antenatal RSV vaccines among postpartum women in Al-Madinah, Saudi Arabia.

**Methods:**

This hospital-based, cross-sectional study was conducted at two hospitals in Al-Madinah, Saudi Arabia. A validated 13-item questionnaire was used, including questions regarding demographic data, awareness of RSV infection, and acceptance of the antenatal RSV vaccine. Multivariate binary logistic regression analysis was used to assess the association between demographic data and awareness and acceptance of the antenatal RSV vaccine.

**Results:**

Among 400 postpartum women (mean age, 30 years; range, 18–44 years), 51% were aware of RSV infection. Most provided correct answers regarding the mode of RSV transmission (72.5%). Approximately 40% of the participants perceived the seriousness of the RSV infection to be high for infants after delivery. Acceptance for receiving the RSV vaccine was moderate (58%). Compared with high school or lower educational level, university or higher education was associated with lower acceptance of the RSV vaccine (odds ratio, 0.72; 95% confidence interval, 0.56–0.93).

**Conclusions:**

Despite moderate awareness of RSV infection, maternal acceptance of the antenatal RSV vaccine among highly educated women was lower than that among women with a lower education level. This low acceptance rate highlights the need for future studies to evaluate the actual uptake of antenatal RSV vaccination among highly educated women.

## 1 Introduction

Respiratory syncytial virus (RSV) is a major cause of acute lower respiratory tract infection (LRTI) in infants and young children. Globally, RSV is responsible for approximately 33 million episodes of LRTI each year among children younger than 5 years, resulting in an estimated more than 100,000 deaths annually (1). According to the Centers for Disease Control and Prevention, RSV causes an estimated 50,000–80,000 hospitalizations and 100–300 deaths annually among children aged <5 years in the United States (2).

Several hospital-based studies conducted in Saudi Arabia between 1991 and 2015 reported that the prevalence of RSV among pediatric patients with acute respiratory infections ranged from 0.2% to 54% (3). Most infections occurred in infants aged <1 year, accounting for 51%–97% of lower respiratory tract infection cases (3). RSV outbreaks typically occur during the winter season in temperate countries, including Saudi Arabia (3). Although the incidence of RSV decreased significantly during the coronavirus disease 2019 (COVID-19) pandemic and associated lockdowns, cases resurged in subsequent years (4).

Until recently, no active immunization against RSV was available, and passive immunization with the monoclonal antibody palivizumab represented the primary preventive strategy. However, its use is restricted to high-risk populations, such as infants born prematurely or those with congenital heart disease or chronic lung disease. In addition, multiple injections are required throughout the RSV season (5).

Maternal vaccination is widely used to reduce the incidence of maternal, neonatal, and infant infections (6). Antibodies transferred transplacentally from mother to fetus provide passive immunity during early infancy (6). A new non-adjuvanted recombinant RSV vaccine (RSVPreF, Abrysvo; Pfizer, New York, NY, USA) was recently approved for use in pregnant women between 32 and 36 weeks of gestation (7, 8). In 2024, several regulatory agencies, including the World Health Organization, recommended its administration during the third trimester of pregnancy (9).

Evidence from several clinical trials has demonstrated that maternal RSV immunization is effective in preventing RSV disease in infants during the first 6 months of life (10). However, few studies have examined factors associated with acceptance or refusal of RSV vaccination among pregnant women in the Middle East (11). In Saudi Arabia, several studies have addressed vaccine hesitancy in general (12), but data specifically related to antenatal RSV vaccine acceptance remain limited.

Maternal RSV vaccination has recently been approved and is currently recommended by public health authorities in Saudi Arabia (13). However, data regarding RSV vaccine acceptance among pregnant women in Saudi Arabia remain scarce. Therefore, this study aimed to assess the awareness of and willingness among women in Al-Madinah, Saudi Arabia, to receive antenatal RSV vaccination.

## 2 Materials and methods

### 2.1 Study design and setting

This hospital-based, cross-sectional study was conducted at two hospitals in Al-Madinah, Saudi Arabia. The study protocol was approved by the institutional review board of King Abdullah International Medical Research Center, Jeddah, Saudi Arabia (Approval No.: NRM24/001/9). Informed consent was obtained from all participants before data collection.

### 2.2 Participants

The study included postpartum women during their first 2 days after delivery in the postnatal wards. Women who did not speak Arabic or English were excluded.

### 2.3 Sampling method

A non-probability sampling method was employed at both study sites. According to a previously published study, 13% of pregnant women were aware of maternal immunization during pregnancy (14). Considering the annual number of deliveries at the study sites, a sample size of 172 participants was required with a 95% confidence level and 5% margin of error. However, doubling the calculated sample size was decided to improve the precision and reliability of the estimates, and to increase the statistical power of the study.

### 2.4 Variables and data collection

Data were collected from pregnant women using a previously published validated questionnaire (15). The questionnaire consisted of 13 items divided into two main sections. The first section collected demographic information, including age, educational level, number of pregnancies and deliveries, nationality, and type of healthcare provider. The second section assessed knowledge and perceptions regarding RSV infection and vaccination, including awareness of RSV infection, perceived severity of RSV infection in infants, knowledge of transmission routes, and acceptance of maternal immunization during pregnancy.

The original questionnaire was first translated into Arabic using a forward-translation process, after which the wording of the items was adapted to reflect the objectives and context of the present study on maternal RSV vaccination at the study sites. The adapted questionnaire was then reviewed by a panel of co-authors with relevant expertise in maternal health, infectious diseases, and research methodology. The experts evaluated the questionnaire for content relevance, clarity, language appropriateness, and contextual suitability for antenatal RSV immunization (supplementary file-1).

Data were collected through face-to-face interviews conducted by trained research team members.

### 2.5 Statistical analysis

Continuous variables with evidence of skewed distribution were summarized using the median and interquartile range (IQR), whereas categorical variables were described using frequencies and percentages. Statistical normality of continuous variables was assessed using the Kolmogorov–Smirnov test and histogram inspection. Multiple-response dichotomy analysis was performed for variables with more than one response option. Multivariable binary logistic regression (MBLR) analysis was used to identify predictors of women's awareness and acceptance of maternal RSV vaccination.

For awareness question (question 10) and acceptance question (question 13), prior to conducting the final analysis, we performed a multinomial logistic regression using the three response categories (“Yes,” “No,” and “I do not know”) to evaluate whether the “I do not know” group differed meaningfully from the “No” group. The multinomial analysis indicated that respondents who selected “I do not know” did not differ significantly from those who responded “No” with respect to the study outcomes and predictor effects. Based on these findings, and to obtain a more parsimonious and interpretable model, the “No” and “I do not know” categories were combined into a single (non-acceptance) category, and binary logistic regression was subsequently performed to compare participants who accepted vaccination (“Yes”) with those who did not accept or were uncertain (“No/I do not know”).

All candidate predictors identified from the literature and considered clinically and theoretically plausible were first examined in bivariate analyses. These results informed the initial selection of variables for multivariable modeling.

All variables were evaluated in the competing multivariable models. Variables that did not demonstrate a statistically significant independent association with the study outcomes after adjustment for other covariates were not retained in the final parsimonious models. Variables demonstrating multicollinearity or contributing minimally to the explanatory power of the models were similarly excluded during the model refinement process.

Associations between predictor variables and study outcomes were expressed as adjusted odds ratios (ORs) with corresponding 95% confidence intervals (CIs). Statistical analyses were performed using IBM SPSS Statistics (IBM Corp., Armonk, NY, USA), and a two-sided alpha level of 0.05 was considered statistically significant.

## 3 Results

### 3.1 Demographic and obstetric characteristics

Among the 400 women who completed the survey, 216 (54%) were aged 25–34 years. The vast majority of participants were Saudi nationals (91.5%). Most participants (40.5%) held a bachelor's degree, whereas only 2.8% (*n* = 11) had attained postgraduate education (i.e., master's or doctoral degree).

Most participants had experienced 2–3 deliveries (44.5%) and were followed primarily by obstetricians during pregnancy (81%). Approximately 20% (*n* = 76) of the participants had high-risk pregnancies. The demographic and obstetric characteristics of the participants are presented in Table 1.

**Table 1:** Sociodemographic and obstetric characteristics of the women (*n* = 400).

Sociodemographic and obstetric characteristic	Frequency (%)
Age group, years	
18–24	46 (11.5)
25–34	216 (54)
35–44	138 (34.5)
Educational level	
Less than high school completed	55 (13.8)
Completed high school	132 (33)
Some post-secondary (e.g., attending college or completed training school)	40 (10)
Completed post-secondary (college degree)	162 (40.5)
Advanced degree (e.g., master's, Doctor of Medicine, Doctor of Philosophy)	11 (2.7)
Nationality	
Non-Saudi	34 (8.5)
Saudi	366 (91.5)
Attended prenatal classes	
Yes	16 (4)
No	384 (96)
Number of pregnancies, median (IQR)	3 (3)
Number of pregnancies	
1	59 (14.8)
2–3	150 (37.5)
4–6	146 (36.5)
≥7	45 (11.3)
Number of deliveries, median (IQR)	3 (1)
Number of deliveries	
1	70 (17.5)
2–3	178 (44.5)
4–6	127 (31.8)
≥7	25 (6.3)
Who is your primary health care provider for this pregnancy?	
Did not have one	7 (1.8)
General practitioner/family doctor	69 (17.2)
Obstetrician	324 (81)
If you were referred to an obstetrician, why were you referred?	
High risk pregnancy	76 (19)
Not referred	324 (75)
Other reasons	24 (6)
Where did you receive prenatal care (ie. Medical care during pregnancy)? You may choose more than one	
Obstetric Clinic-National Guard (NG) Hospital	40 (10.2)
Primary Health Care Center -NG health affairs	12 (3.1)
Obstetric Clinic-Ohud hospital	7 (8.1)
Primary health care center-Ministry of health	107 (27.2)
Obstetric clinic- Madinah Maternity and children hospital (MMCH)	160 (40.7)
Private sector	200 (50)

IQR, interquartile range.

### 3.2 Awareness and vaccine acceptance

Approximately half of the participants (51%) were aware of RSV infection, and nearly 40% perceived RSV infection as a major threat to infants after birth. The majority of participants correctly identified the mode of transmission.

Regarding acceptance of antenatal RSV vaccination, 58% of participants expressed willingness to receive the vaccine, 23.5% declined vaccination, and 18.5% were uncertain. Responses related to RSV awareness and vaccine acceptance are summarized in Table 2.

**Table 2:** Responses to the questions on awareness and acceptance of the antenatal RSV vaccine (*n* = 400).

Awareness and acceptance questions	Frequency (%)
Before receiving the study information form, had you heard of RSV infection?	
I do not know	3 (0.8)
No	193 (48.3)
Yes	204 (51)
Which of the following statements do you believe to be true about RSV infection?	
Do not know	88 (22)
RSV is a sexually transmitted disease	4 (1)
RSV can be passed to the fetus during pregnancy	18 (4.5)
Newborns get RSV mainly from their mother or other family members after birth	290 (72.5)
How serious of a threat do you think RSV poses to a baby after delivery?	
Not sure	124 (31)
Low	30 (7.5)
Moderate	87 (21.8)
High	159 (39.8)
Would you want to receive the RSV vaccine if it is offered to you?	
I do not know	74 (18.5)
No	94 (23.5)
Yes	232 (58)

RSV, respiratory syncytial virus.

### 3.3 Association between participants’ characteristics and awareness of RSV infection

In the MBLR analysis, no statistically significant association was observed between demographic or obstetric characteristics and awareness of RSV infection. However, women who perceived RSV infection as posing a moderate-to-high risk to infants were significantly more likely to be aware of RSV infection (OR, 3.19; 95% CI, 1.72–5.91). The results of the MBLR analysis for RSV awareness are presented in Table 3.

**Table 3:** Multivariate binary logistic regression analysis results of women's awareness of RSV infection.

Variables	Multivariate-adjusted odds ratio	95% CI		*p*-value
		Lower	Upper	
Age group	0.86	0.53	1.37	0.53
Number of pregnancies	1.01	0.88	1.16	0.85
Educational level: College (University) degree or higher vs. lower education	0.79	0.63	1.00	0.05
Nationality	0.81	0.31	2.12	0.67
Type of health care provider	0.75	0.45	1.26	0.28
Perceives high to moderate threat of RSV for infants Vs low threat	3.19	**1**.**72**	**5**.**91**	**<0**.**001**
Constant	1.14			0.92
Dependent outcome variable: before receiving the study information form, had you heard of RSV infection? No/Yes				

RSV, respiratory syncytial virus, CI: confidence interval.

Bold values indicate statistically significant associations, defined as P < 0.05 (equivalently, a 95% confidence interval that does not include 1.0).

### 3.4 Association between participants’ characteristics and acceptance of the RSV vaccine

Regarding acceptance of antenatal RSV vaccination, the MBLR analysis demonstrated that women with higher educational attainment (i.e., university education or above) were less likely to accept the antenatal RSV vaccine compared with women with lower educational levels (OR, 0.72; 95% CI, 0.56–0.93). None of the remaining demographic or obstetric characteristics showed a statistically significant association with acceptance or refusal of the RSV vaccine (Table 4).

**Table 4:** Multivariate binary logistic regression analysis results of women's acceptance of the RSV vaccine.

Variables	Multivariate-adjusted OR	95% CI for OR		*p*-value
		Lower	Upper	
Age group	0.80	0.48	1.34	0.41
Educational level: College (University) degree or higher vs. lower education	0.72	**0**.**56**	**0**.**93**	**0**.**01**
Number of pregnancies	0.98	0.84	1.13	0.78
Nationality	0.72	0.28	1.81	0.48
Perceives a high to moderate threat of RSV for the infant Vs low threat	1.18	0.95	1.46	0.12
Constant	0.37			0.33
Dependent outcome variable: would you want to be immunized if it was offered to you? No/Yes				

OR, odds ratio, CI, confidence interval.

Bold values indicate statistically significant associations, defined as P < 0.05 (equivalently, a 95% confidence interval that does not include 1.0).

## 4 Discussion

The present study showed that approximately half of the pregnant women were unaware of RSV infection before participating in the study. However, the number of studies evaluating women's awareness of RSV infection and antenatal RSV vaccines has increased in recent years. Gavaruzzi et al. conducted a recent systematic review assessing knowledge and attitudes toward RSV prevention across different populations (16). In most studies included in the review, the proportion of pregnant women unaware of RSV infection ranged from 20% to 70% (16).

Compared with women from other countries in the region, participants in our study demonstrated lower awareness of RSV infection (51%) than pregnant women in Jordan (67.6%) (17). This difference may be attributed to variations in educational attainment. Approximately 53% of participants in our study were currently enrolled in or had completed a college degree, compared with approximately 90% in the Jordanian study (17).

Approximately 60% of women in our study perceived RSV infection as posing a moderate or high threat to their infants. This proportion is substantially higher than that reported in an Australian study, in which only approximately 10% of women perceived RSV as a serious infection in infants (18). Notably, 90% of Australian participants were unaware of the seriousness of RSV infection in infants, compared with 31% in our study (18). In contrast, another study reported a higher perceived severity of RSV infection (19). In a multinational survey involving 5,627 parents of children aged <24 months, >90% perceived RSV infection as posing a moderate or high risk to infants (19). In our study, women who perceived RSV infection as a moderate or high risk to infants were significantly more likely to be aware of RSV infection than those who perceived it as low risk (OR, 3.197; *p* < 0.001). This finding may reflect prior personal or family experience with RSV infection.

More than two-thirds of participants correctly identified the mode of RSV transmission. Data regarding general knowledge of RSV infection among pregnant women remain limited. In a study conducted in Nepal, 95% of participants were aware of the association between RSV infection and bronchiolitis (20). Similarly, a study from Poland reported that approximately 90% of participants were aware of RSV symptoms (21). However, this proportion declined to approximately 50% for questions related to RSV transmission and treatment (21).

Approximately half of the women in our study expressed willingness to receive the RSV vaccine during pregnancy. Previous studies evaluating acceptance of routine maternal RSV vaccination have reported acceptance rates ranging from 42% to 88% (16). The acceptance rate observed in our study falls within this previously reported range.

In the present study, higher educational attainment (university level or above) was associated with lower acceptance of RSV vaccination. This finding contrasts with results from a previous study conducted in Jordan, in which university education was associated with greater odds of vaccine acceptance compared with a high school education or lower, with ORs of 3.26 (95% CI, 1.15–9.27) and 0.97 (95% CI, 0.28–3.36), respectively (11).

Limited awareness regarding the availability and recommendations for routine antenatal vaccinations, such as influenza and pertussis vaccines, may contribute to lower acceptance of maternal vaccination in general. A study conducted in Riyadh, Saudi Arabia, involving 998 pregnant women, reported low awareness (13%) and uptake (19.1%) of influenza vaccination during pregnancy (22). Although uptake of other antenatal vaccines was not assessed in our study, several studies have shown that prior acceptance of maternal vaccinations, such as influenza vaccination, is associated with increased acceptance of RSV vaccination during pregnancy (11, 23). In a study from the United States, women who had received antenatal vaccination during a previous pregnancy had approximately threefold higher odds of accepting RSV vaccination compared with women who had not received antenatal vaccines previously (OR, 3.56; *p* < 0.001) (23).

The relationship between educational level and vaccine acceptance during pregnancy has been explored in several studies. In general, higher educational attainment has been associated with greater vaccine acceptance (16). However, some studies have reported findings similar to those of the present study, in which higher educational attainment was associated with lower vaccines acceptance. For example, Wang et al. reported that women with university-level education or higher were less willing to accept RSV vaccination (OR, 0.51; 95% CI, 0.31–0.83), whereas lower educational attainment was not significantly associated with vaccine acceptance (OR, 0.93; 95% CI, 0.60–1.44) (24). Similarly, a study conducted in Riyadh, Saudi Arabia, examining parental vaccine hesitancy found that higher educational attainment was significantly associated with vaccine hesitancy (*p* < 0.001) (12).

These inconsistent findings regarding the association between educational attainment and vaccine acceptance may be explained by additional factors, including socioeconomic determinants. A recent study evaluated the relationship between vaccine acceptance and national economic indicators using components of the Human Development Index, including education level (25). Based on national COVID-19 vaccination uptake data, higher educational attainment was generally associated with higher vaccination rates. However, multivariable regression analysis revealed that in high-income countries, increased educational expenditure was associated with higher vaccine uptake, whereas in lower-income countries, the relationship was inverse (25).

This study has several limitations. First, the survey instrument may not have captured all potential predictors of vaccine acceptance, highlighting the need for future qualitative research to explore additional factors influencing maternal RSV vaccine acceptance. Furthermore, the maternal RSV vaccine had only recently been approved in Saudi Arabia at the time of the study, and its widespread implementation in clinical practice had not yet occurred. Consequently, participants’ perceptions may not fully reflect attitudes toward a routinely available vaccine. Future studies conducted following broader implementation of the maternal RSV vaccination program are warranted to better assess predictors of vaccine uptake and acceptance.

Second, the study was conducted in a single city, which may limit the generalizability of the findings to the wider Saudi population. Despite these limitations, to our knowledge, this is the first study to evaluate awareness and acceptance of antenatal RSV vaccination among women in Saudi Arabia, providing important baseline data to inform future research and public health strategies.

## 5 Conclusions

The acceptance rate of antenatal RSV vaccination among pregnant women in Al-Madinah, Saudi Arabia, was within the range reported internationally. However, women with higher educational attainment demonstrated lower vaccine acceptance compared with those with lower educational levels. Further qualitative research is needed to explore the underlying reasons for this hesitancy and to inform public health policies related to the implementation of antenatal RSV vaccination programs.

## Data Availability

The original contributions presented in the study are included in the article/Supplementary Material, further inquiries can be directed to the corresponding author.
