# Supplementary material for: Association between educational level and acceptance of antenatal respiratory syncytial virus vaccination among Saudi women: a cross-sectional study
Source: Front Pediatr. 2026 Jul 17;14:1900895. doi: 10.3389/fped.2026.1900895 (PMC13424492; doi:10.3389/fped.2026.1900895)
Supplement: Supplementary file 1 [file Datasheet1.pdf]

Knowledge, Attitude and Practices of pregnant women toward maternal immunization (**Respiratory syncytial virus- RSV**) at Al-Madina

First part: Demographics

1.What is your age?

a. Under 18
b.18-24
c.25-34
d.35-44
e.45 or older

2.What is your highest level of education?
a. Less than high school completion
b. Completed high school
c. Some post-secondary (e.g. undergoing college, or completed training school)
d. Completed post-secondary (college degree- University)
e. Advanced degree (e.g. master, Doctor of Medicine, Doctor of Philosophy)

3.How many pregnancies have you had?

4.How many deliveries have you had?

5.What is your nationality?

a. Saudi
b. non-Saudi

6.Did you attend prenatal classes?

a. Yes
b. No

7.Who is your primary health care provider for this pregnancy? You may choose more than one

a. GP (General Practitioner/ Family Doctor)
b. Obstetrician
c. Other (specify): ___________________
d. Not referred

8.If you were referred to an obstetrician, why were you referred?
a. High risk pregnancy
b. Other (specify): _____
c. N/A

9.Where did you receive prenatal care (ie. Medical care during pregnancy)? You may choose more than one

a. Primary health care center-Ministry of health (PHC MOH)
b. Primary Health Care Center (PHC)-National guard health affairs
c. Obstetric clinic- Prince Mohammed Bin Abdulaziz Hospital, NGHA
d. Obstetric clinic- Madinah Maternity and children hospital (MMCH)
e. private sector

Respiratory syncytial virus (RSV):

10. Before receiving the study information form, had you heard of RSV infection?

a. Yes
b. No
c. Don't know

11. Which of the following statements do you believe to be true about RSV infection?

A. RSV is a sexually transmitted disease
B. RSV can be passed to the fetus during pregnancy

C. **<u>Newborns get RSV mainly from their mother or other family members after birth</u>**
D. Do not know

12.**How serious a threat do you think RSV poses to a baby after delivery?**

A. High
B. Moderate
C. Low
D. Not sure

13. At present, RSV vaccine is recommended during pregnancy but still not available at the Kingdom of Saudi Arabia. If it were known that you could possibly protect your newborn in the first months of life by being immunized during pregnancy (and there was no known risk for you or your baby), would you be interested in being immunized during pregnancy?

a. Yes
b. No
c. Don't know
